# Supplementary material for: High-fidelity neural speech reconstruction through an efficient acoustic-linguistic dual-pathway framework
Source: eLife. 2026 Mar 5;14:RP109400. doi: 10.7554/eLife.109400 (PMC12962650; doi:10.7554/eLife.109400)
Supplement: Supplementary file 1. — Performance is evaluated by the mel-spectrogram R² (mean ± s.e.m. across participants), measuring the fidelity of reconstructed acoustic features. The bidirectional long-short term memory (LSTM) adaptor consistently outperformed the Transformer-based adaptor across different layer depths. The optimal performance was achieved with a 3-layer LSTM, which was selected for the final model. [file elife-109400-supp1.docx]

| **Adaptor** | **Layers** | **Mel-spectrogram R^2^** |
| --- | --- | --- |
| LSTM | 1 | 0.780 ± 0.016 |
| LSTM | 2 | 0.788 ± 0.016 |
| LSTM | 3 | 0.793 ± 0.016 |
| LSTM | 4 | 0.789 ± 0.015 |
| LSTM | 5 | 0.792 ± 0.015 |
| Transformer | 1 | 0.759 ± 0.016 |
| Transformer | 2 | 0.761 ± 0.016 |
| Transformer | 3 | 0.761 ± 0.017 |
| Transformer | 4 | 0.757 ± 0.017 |
| Transformer | 5 | 0.727 ± 0.014 |

**Supplementary File 1. Ablation study on adaptor architecture for the acoustic pathway.**
Performance is evaluated by the mel-spectrogram R² (mean ± s.e.m. across participants), measuring the fidelity of reconstructed acoustic features. The bidirectional LSTM adaptor consistently outperformed the Transformer-based adaptor across different layer depths. The optimal performance was achieved with a 3-layer LSTM, which was selected for the final model.
