# Supplementary material for: High-fidelity neural speech reconstruction through an efficient acoustic-linguistic dual-pathway framework
Source: eLife. 2026 Mar 5;14:RP109400. doi: 10.7554/eLife.109400 (PMC12962650; doi:10.7554/eLife.109400)
Supplement: Supplementary file 2. — Performance is evaluated by word error rate (WER) and phoneme error rate (PER) (mean ± s.e.m. across participants), measuring the intelligibility of reconstructed speech. The Transformer-based adaptor achieved lower error rates than the long-short term memory (LSTM)-based adaptor across nearly all layer configurations. The optimal performance was achieved with a 3-layer Transformer, which was selected for the final model. [file elife-109400-supp2.docx]

| **Adaptor** | **Layers** | **WER (%)** | **PER (%)** |
| --- | --- | --- | --- |
| LSTM | 1 | 28.0 ± 4.0 | 15.9 ± 2.7 |
| LSTM | 2 | 26.7 ± 4.0 | 15.0 ± 2.7 |
| LSTM | 3 | 25.0 ± 3.2 | 14.1 ± 2.5 |
| LSTM | 4 | 24.8 ± 3.6 | 14.0 ± 2.5 |
| LSTM | 5 | 25.7 ± 4.0 | 14.4 ± 2.6 |
| Transformer | 1 | 22.5 ± 3.8 | 12.7 ± 2.4 |
| Transformer | 2 | 19.9 ± 3.4 | 11.6 ± 2.3 |
| Transformer | 3 | 17.7 ± 3.2 | 11.0 ± 2.3 |
| Transformer | 4 | 19.9 ± 3.4 | 11.5 ± 2.3 |
| Transformer | 5 | 21.3 ± 3.6 | 12.2 ± 2.3 |

**Supplementary File 2. Ablation study on adaptor architecture for the linguistic pathway.**
Performance is evaluated by word error rate (WER) and phoneme error rate (PER) (mean ± s.e.m. across participants), measuring the intelligibility of reconstructed speech. The Transformer-based adaptor achieved lower error rates than the LSTM-based adaptor across nearly all layer configurations. The optimal performance was achieved with a 3-layer Transformer, which was selected for the final model.
