# Supplementary material for: High-fidelity neural speech reconstruction through an efficient acoustic-linguistic dual-pathway framework
Source: eLife. 2026 Mar 5;14:RP109400. doi: 10.7554/eLife.109400 (PMC12962650; doi:10.7554/eLife.109400)
Supplement: Supplementary file 3. — The table summarizes representative work, highlighting the neural recording modality, approximate amount of data used for decoder training per subject, the primary experimental task (perception or production), and reported performance metrics. Studies are ordered chronologically. Performance metrics include: Mean Opinion Score (MOS, scale 1–5), Extended Short-Time Objective Intelligibility (ESTOI, scale 0–1), Word Error Rate (WER, %), Phoneme Error Rate (PER, %), and mel-spectrogram correlation (R², scale 0–1). Note that direct numerical comparisons should be made with caution due to differences in neural signals, tasks, stimuli, and evaluation methodologies across studies. Our study (highlighted in bold) achieves a competitive balance between data efficiency (~20 min) and performance across multiple metrics (WER, PER, MOS, R²). [file elife-109400-supp3.docx]

| **Authors** | **Year** | **Neural recording modality** | **Neural recording durations** | **Primary task** | **Decoding framework** | **Performance** |
| --- | --- | --- | --- | --- | --- | --- |
| Akbari et al. *(1)* | 2019 | ECoG | 30 minutes | Speech perception | CNN + Vocoder | MOS=3.4  0.35 < ESTOI < 0.40 |
| Komeiji et al. *(2)* | 2022 | ECoG | 10-15 minutes | Speech production | CNN + Transformer + LSTM | PER = 31.3% |
| Bellier et al. *(3)* | 2023 | ECoG | 190.72 seconds | Music perception | MLP | Spectrogram R^2^ = 0.429 |
| Willett et al. *(4)* | 2023 | Utah array | Approximately 100 hours | Speech production | GRU | WER = 24.7%  PER = 20.9% |
| Metzger et al. *(5)* | 2023 | ECoG | 20.8 hours | Speech production | RNN + pre-trained speech encoder | WER = 25.5% |
| Li et al. *(6)* | 2024 | ECoG | 20 minutes | Speech perception | LSTM + pre-trained speech decoder | ESTOI = 0.371  PER = 28.6%  MOS = 2.9 |
| Chen et al. *(7)* | 2024 | ECoG | 200 seconds | Speech perception and production | ECoG decoder + pre-trained speech synthesizer | Spectrogram R^2^ = 0.81 |
| Wairagkar et al. *(8)* | 2025 | Utah array | More than 400 days | Online speech production | Transformer + TTS model | Spectrogram R^2^ = 0.83  WER = 45.8%  PER = 34.0% |
| Li et al. (Ours) | 2025 | ECoG | 20 minutes | Speech perception | Acoustic and linguistic pathways (adaptor + generator) + voice cloner | Mel-spectrogram R^2^ = 0.82  MOS = 4.0  WER = 18.9%  PER = 12.0% |

**Supplementary File 3. Comparative overview of recent studies in neural-driven speech decoding and re-synthesis.**

The table summarizes representative work, highlighting the neural recording modality, approximate amount of data used for decoder training per subject, the primary experimental task (perception or production), and reported performance metrics. Studies are ordered chronologically. Performance metrics include: Mean Opinion Score (MOS, scale 1-5), Extended Short-Time Objective Intelligibility (ESTOI, scale 0-1), Word Error Rate (WER, %), Phoneme Error Rate (PER, %), and mel-spectrogram correlation (R², scale 0-1). Note that direct numerical comparisons should be made with caution due to differences in neural signals, tasks, stimuli, and evaluation methodologies across studies. Our study (highlighted in bold) achieves a competitive balance between data efficiency (~20 minutes) and performance across multiple metrics (WER, PER, MOS, R²).
